# Habitat heterogeneity and connectivity shape microbial communities in South American peatlands

**DOI:** 10.1038/srep25712

**Published:** 2016-05-10

**Authors:** Felix Oloo, Angel Valverde, María Victoria Quiroga, Surendra Vikram, Don Cowan, Gabriela Mataloni

**Affiliations:** 1 Centre for Microbial Ecology and Genomics (CMEG), Department of Genetics, University of Pretoria, Pretoria, South Africa; 2 Instituto de Investigaciones Biotecnológicas–Instituto Tecnológico de Chascomús (IIB-INTECH), Universidad Nacional de San Martín - Consejo Nacional de Investigaciones Científicas y Técnicas, Argentina; 3 Instituto de Investigación e Ingeniería Ambiental (3iA), Universidad Nacional de San Martín, Buenos Aires, Argentina

## Abstract

Bacteria play critical roles in peatland ecosystems. However, very little is known of how habitat heterogeneity affects the structure of the bacterial communities in these ecosystems. Here, we used amplicon sequencing of the 16S rRNA and *nifH* genes to investigate phylogenetic diversity and bacterial community composition in three different sub-Antarctic peat bog aquatic habitats: *Sphagnum magellanicum* interstitial water, and water from vegetated and non-vegetated pools. Total and putative nitrogen-fixing bacterial communities from *Sphagnum* interstitial water differed significantly from vegetated and non-vegetated pool communities (which were colonized by the same bacterial populations), probably as a result of differences in water chemistry and biotic interactions. Total bacterial communities from pools contained typically aquatic taxa, and were more dissimilar in composition and less species rich than those from *Sphagnum* interstitial waters (which were enriched in taxa typically from soils), probably reflecting the reduced connectivity between the former habitats. These results show that bacterial communities in peatland water habitats are highly diverse and structured by multiple concurrent factors.

Bacterial communities in peat bog ecosystems contribute significantly to nutrient cycling, to carbon sequestration and to greenhouse gas emissions^1^,^2^,^3^,^4^. However, we still have a limited knowledge of the diversity and spatial distribution of bacterial assemblages in peat bogs. As a result, we do not know, for example, how the composition of bacterial communities and their traits differ across peatland water bodies; although recent work has shown that bacterioplankton communities in peat bog pools are diverse and variable^5^. Understanding the diversity and spatial distribution of microbial communities across different peatland aquatic habitats is important, because these habitats are highly heterogeneous^6^; and habitat heterogeneity can lead to changes in biodiversity patterns, which provide information to many ecological and evolutionary questions, as well as to conservation planning^7^. The latter is especially crucial, as peat bog ecosystems may be highly vulnerable to climate change^8^.

Habitat differences between peatland water bodies may arise for a number of reasons. For example, peatlands can have contrasting nutrient conditions^9^, and it is well known that microbes often differ in their nutritional requirements^10^. Copiotrophic microbes have high nutritional requirements, while oligotrophic microbes are likely to outcompete copiotrophs in conditions of low nutrient availability^10^. Environmental parameters such as pH^5^ and DOC^11^ may also be important in determining microbial community composition in peatlands. The role of aquatic vegetation cannot be ignored either. The lack of surface vegetation allows the penetration of high levels of photosynthetically active radiation (PAR) and ultraviolet radiation, with consequences for nutrient cycling and primary production, which in turn, can promote compositional differences in microbial communities^12^. Furthermore, peatland vegetation is often dominated by *Sphagnum* mosses, and different *Sphagnum* species harbour different bacterial populations^13^.

Peatland water bodies also differ in connectivity, which influences microbial diversity patterns^14^,^15^,^16^. For instance, the total number of species in habitat patches connected by moderate dispersal should be higher than in a single large patch^17^. In addition to their effects on species richness, interconnected habitats may favour species with generalist traits. This should lead to a decline in community turnover because of increased homogenization of the metacommunity^18^. Consequently, understanding how habitat heterogeneity and differences in connectivity affect the distribution of bacterial communities and their traits is important to better understand the structure-function relationship in peat bog ecosystems and for conservation planning.

Here, we analysed the total and nitrogen-fixing bacterial communities found in three different aquatic habitats (vegetated and clear pools, and *Sphagnum magellanicum* interstitial water) within two peat bog complexes (Rancho Hambre and Valle de Andorra, Tierra del Fuego, Argentina), using high-throughput DNA sequencing of 16S rRNA and *nifH* genes. Biological nitrogen fixation of atmospheric nitrogen is an important process in peatlands, because the growth of *Sphagnum* mosses in peatlands is often N limited^19^. Vegetated and clear pools can be regarded as nutrient-poor island-like habitats embedded in a landscape of *Sphagnum* and fed essentially by rainfall, whereas *Sphagnum* interstitial waters are less nutrient-poor and exhibit a major degree of connectivity and are further linked to the surrounding landscape. Thus, we predict that the structure of both total bacterial and putative nitrogen-fixing communities will reflect the striking differences in nutrient status and connectivity between these habitats.

## Results and Discussion

**Figure 1:**
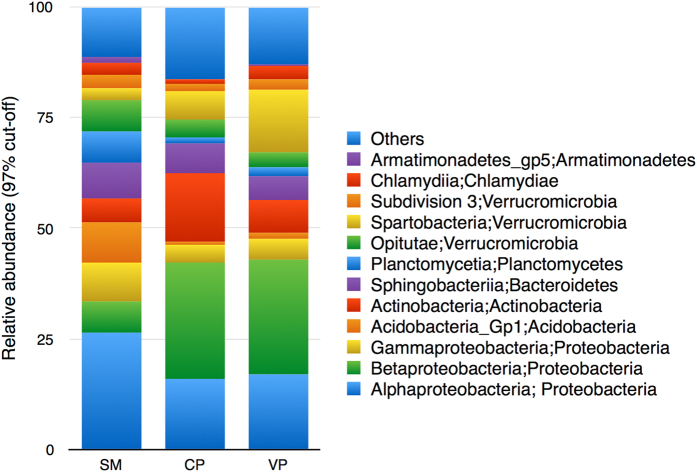
Taxonomic information (class;phylum) based on 16S rRNA gene sequences (classified with confidence threshold of 80%) and expressed as fraction of total sequences. The group Other encompasses unclassified sequences together with classes representing ≤1% of total sequences. CP, clear pools; VP, vegetated pools; SM, *S. magellanicum* interstitial waters.

Rarefaction plots, Chao1 and Good’s coverage (95–100%) estimates indicated reasonable sequencing coverage, especially for *nifH* genes, (Supplementary Table S1, Supplementary Fig. S1); although more sequencing depth would be required to assess the true diversity of the samples. Overall, total bacteria and nitrogen-fixing taxa from *Sphagnum* interstitial waters were more diverse than those from pools (Supplementary Table S1, Supplementary Fig. S2). However, the differences were not statistically significant for putative nitrogen-fixing taxa. Most of the bacterial taxa found were members of the *Proteobacteria*, *Verrucomicrobia*, *Actinobacteria*, *Bacteroidetes*, *Acidobacteria* and *Planctomycetes* (Fig. 1), all of which are commonly found in peatland ecosystems^1^,^5^,^20^,^21^. Nevertheless, *Betaproteobacteria* and *Actinobacteria* dominated pool communities, while *Sphagnum* interstitial water contained a larger proportion of *Alphaproteobacteria*, *Acidobacteria and Planctomycetes*. This is in agreement with previous studies showing that members of these phyla are commonly found in association with *Sphagnum* mosses^13^,^22^.

**Figure 2:**
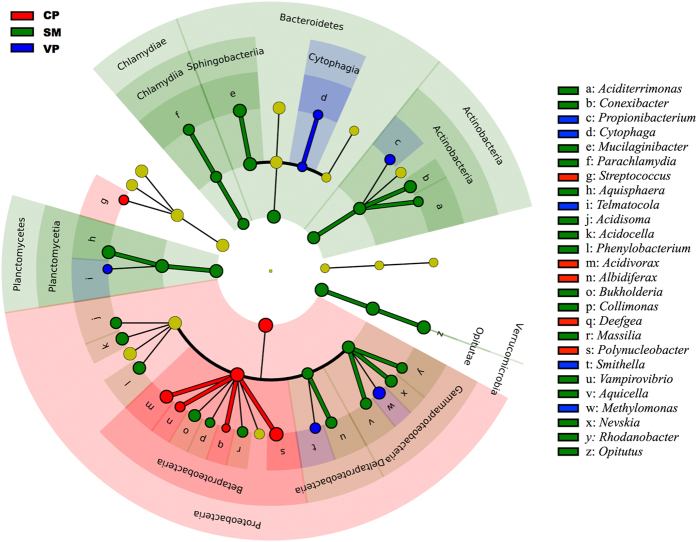
Least discriminant analysis (LDA) effect size taxonomic cladogram, based on 16S rRNA gene sequences, comparing all samples for the three habitats. Significantly discriminant taxon nodes are coloured and branch areas are shaded according to the highest ranked group for that taxon. If the taxon is not significantly differentially represented among sample habitats, the corresponding node is coloured yellow.

The differences in composition were also evident at the genus level. LEfSe analysis identified 26 bacterial genera as biomarkers (Fig. 2). *Sphagnum* interstitial water samples were enriched in genera such as *Aciditerrimonas* (*Actinobacteria*), *Mucilaginibacter* (*Bacteroidetes*) and *Acidisoma* (*Alphaproteobacteria*), whereas *Propionibacterium* (*Actinobacteria*), *Polynucleobacter* (*Betaproteobacteria*) and *Methylomonas* (*Gammaproteobacteria*) were overrepresented in pools. We do not have a complete understanding about the specific ecology of these microorganisms in peatland ecosystems. However, members of the genus *Aciditerrimonas* and *Methylomonas* have been reported as *Sphagnum*-associated methanotrophs^4^,^23^, which are an important sink for the methane produced in peatlands^24^. *Mucilaginibacter* spp. has the ability to use a broad range of heteropolysaccharides, such as xylan, laminarin, or pectin, in acidic and cold conditions^1^. Several bacterial strains affiliated to *Acidisoma* (e.g. *A. sibiricum* and *A. tundrae*) are aerobic, acidophilic, peat-inhabiting chemo-organotrophs that utilize a variety of sugars, polyalcohols and polysaccharides^25^. Fermenting bacteria such as *Propionibacterium* can degrade carbohydrates and polymeric compounds, therefore participating in carbon turnover^26^. Members of the genus *Polynucleobacter* can perform the assimilatory reduction of nitrate and assimilate sulphur and sulphate^27^. In regard to putative nitrogen-fixing microbes, most sequences were related to *Proteobacteria*, *Firmicutes*, *Cyanobacteria* and *Verrucomicrobia* (Supplementary Fig. S3). At the genus level, sequences closely related to *Methylobacter* (*Gammaproteobacteria*) and *Desulfobulbus* (*Deltaproteobacteria*) species dominated in pools, while sequences related to *Bradyrhizobium* (*Alphaproteobacteria*) and *Burkholderia* (*Betaproteobacteria*) species were overrepresented in *Sphagnum* interstitial waters. The *nifH* deduced amino acid sequences showed ≥96% similarity in BLAST analysis in all cases. Noteworthy, both *Bradyrhizobium* and *Burkholderia* species have been previously reported to associate with *Sphagnum* mosses^28^. In fact, *Burkholderia* strains have been found to be transmitted by *Sphagnum* mosses over their life cycle, highlighting the relevance of this association^29^. In all, our findings as well as others^19^,^28^,^30^, suggest that nitrogen fixation may be important in facilitating plant growth under ombrotrophic, nitrogen-limited conditions in bog ecosystems.

**Figure 3:**
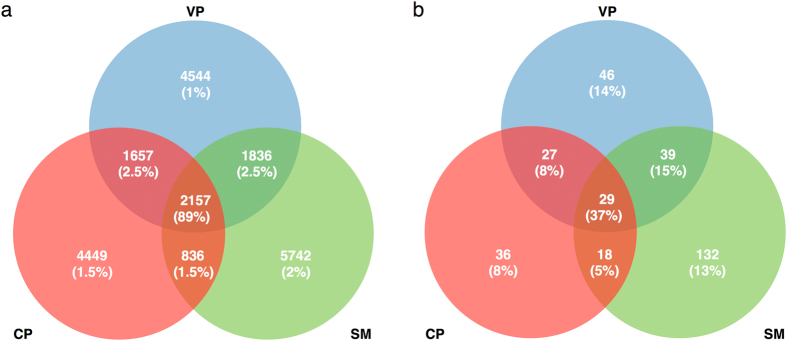
Venn diagram showing the number of (**a**) shared total bacterial and (**b**) nitrogen-fixing OTUs. The percentage of sequences associated with OTUs is shown in parentheses. CP, clear pools; VP, vegetated pools; SM, *S. magellanicum* interstitial waters.

**Figure 4:**
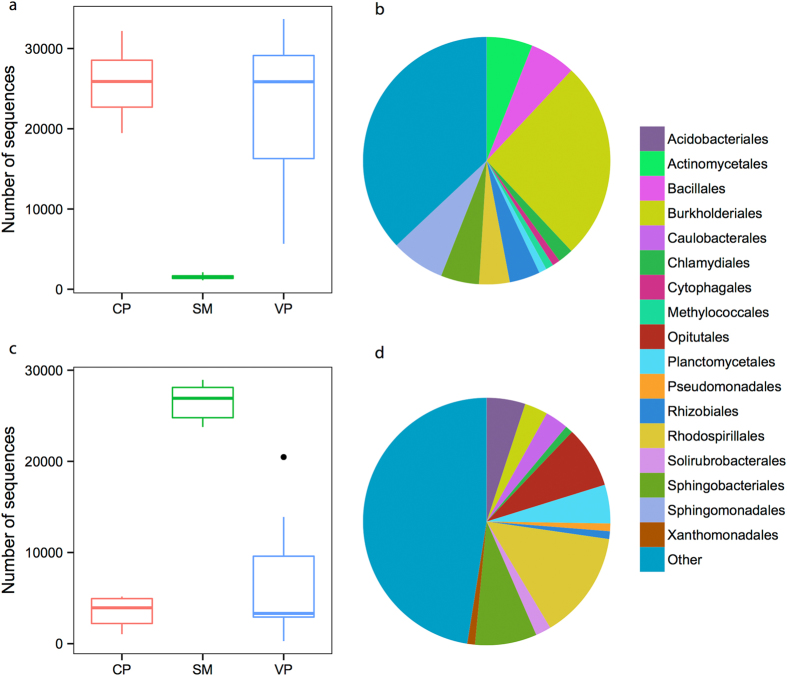
Taxonomic composition and abundance patterns of the two groups of 16S rRNA-derived OTUs that showed the largest changes in abundance between *Sphagnum* interstitial water and pools samples. The group Other encompasses unclassified sequences together with orders representing ≤1% of total sequences. (**a**,**b**) OTUs that dominated pools (n = 169). (**c**,**d**) OTUs that dominated *S. magellanicum* interstitial waters (n = 200). Taxonomic data are presented as per cent contribution of each bacterial order to total sequences. OTUs were selected following the procedure described by Ruiz-González *et al*.^15^ using a mean distance >15. CP, clear pools; VP, vegetated pools; SM, *S. magellanicum* interstitial waters.

**Figure 5:**
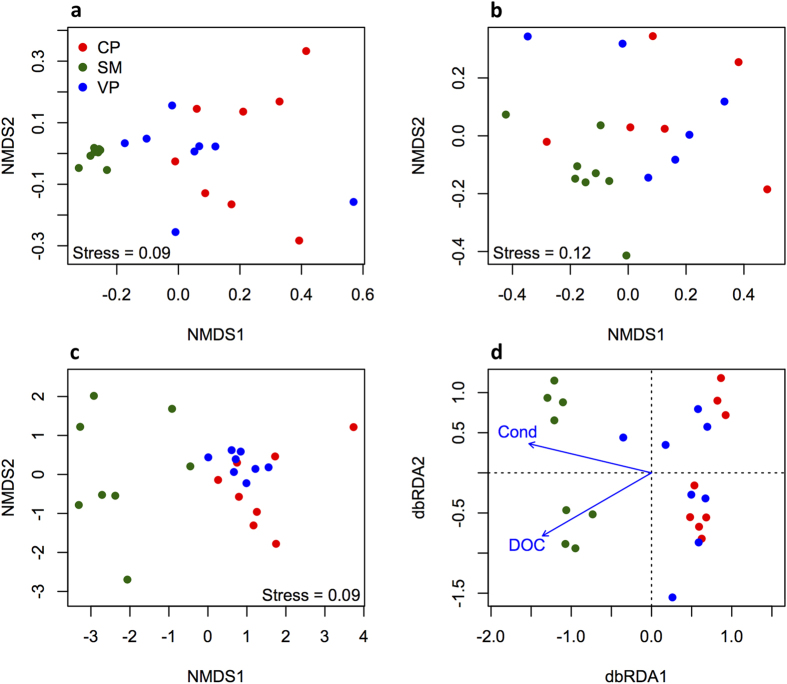
Multidimensional scaling diagrams showing the degree of similarity (Bray-Curtis index) between (**a**) total bacterial communities, (**b**) putative nitrogen-fixing communities and (**c**) environmental conditions. (**d**) Redundancy analysis (RDA) biplot of total bacterial diversity and microenvironmental parameters. Only the environmental variables that significantly explained variability in microbial community structure are fitted to the ordination (arrows). The direction of the arrows indicates the direction of maximum change of that variable, whereas the length of the arrow is proportional to the rate of change. CP, clear pools; VP, vegetated pools; SM, *S. magellanicum* interstitial waters.

Relatively few bacterial OTUs were shared between all three communities (Fig. 3), but these shared OTUs accounted for 89% and 37% of the sequences for total and nitrogen-fixing bacterial communities, respectively. Interestingly, a large proportion of the taxa abundant in *Sphagnum* interstitial waters were rare in pools and *vice versa* (Fig. 4), suggesting a significant degree of habitat preference. Consequently, total bacterial communities, using Bray-Curtis dissimilarities, were found to differ in composition between *Sphagnum* interstitial water and pool samples (PERMANOVA: F_2,23_ = 3.43, R^2^ = 0.25, P = 0.001; Fig. 5a), but not between vegetated and clear pools (P > 0.5). Similar findings were obtained using weighted UniFrac dissimilarities (Supplementary Fig. S4a). Putative nitrogen-fixing bacterial communities gave comparable results to those of the total bacteria (Mantel r = 0.65, P = 0.001) (Fig. 5b, Supplementary Fig. S4b). Interestingly, total bacterial communities were considerably less variable in composition in *Sphagnum* interstitial water samples than in pool samples (Fig. 5a, Supplementary Figs S4a and S5a), which is interpreted as a sign of biotic homogenization. In contrast, nitrogen-fixing bacterial communities were equally variable between the three habitats (Fig. 5b, Supplementary Fig. S4b).

When differences in water chemistry were included in a non-metric multidimensional scaling ordination plot, *Sphagnum* interstitial water and pool samples grouped separately (PERMANOVA: F_2,23_ = 8.9, R^2^ = 0.45, P = 0.001; Fig. 5c). Conversely, no differences were found between vegetated and clear pools (P > 0.05). In addition, permutation dispersion showed that vegetated pools were more similar in chemical composition than *Sphagnum* interstitial water and clear pools (Supplementary Fig. S5b). A *post-hoc* ANOVA analysis found *Sphagnum* interstitial water samples having higher values of, phosphate, DOC and Chl *a* than pools (Supplementary Fig. 6). Using distance-based redundancy analysis we found that DOC and Conductivity explained 16% and 6% (P = 0.001) of the variation in total (Fig. 5d) and nitrogen-fixing (not shown) bacterial communities, respectively.

We propose three non-mutually exclusive explanations for the observed patterns in community composition, related to differences in (1) abiotic environmental conditions, (2) biotic interactions and (3) connectivity. Firstly, the fact that bacterial communities were different in the *Sphagnum* interstitial waters and the pools indicates a strong habitat effect, which is consistent with the concept of species sorting (e.g., ref. 31). Conductivity, a proxy for salinity, and DOC were the dominant drivers of the total bacterial communities (Fig. 5d). Previous studies have identified DOC to be a key driver for aquatic microorganisms^32^, and it is well established that different aquatic bacterial taxa have different preferences in terms of carbon utilization^33^. Similarly, salinity has been found to greatly influence bacterial diversity in aquatic ecosystems^34^, although to our knowledge, this is the first time it has been reported in peatland water bodies.

Secondly, only 6–16% of the total variation in bacterial community composition could be explained by conductivity and DOC, suggesting that other abiotic or biotic factors may be of greater importance^35^. For example, it is well known that *Sphagnum* mosses are colonized by species-specific microbial populations, which fulfil important functions (e.g. nutrient supply and pathogen defence) for moss growth and health^13^,^30^. For instance, *Sphagnum fallax* is colonized mainly by *Verrucromicrobia* and *Planctomycetes*, while *Sphagnum magellanicum* is dominated by *Alphaproteobacteria* and *Gammaprotobacteria*^13^. Both phyla were well represented in *Sphagnum* interstitial water samples (Fig. 1). Zooplankton, protists and viruses could also have a role in structuring these communities^36^. Different bacterial taxa are known to vary in their resistance to both grazing and viral lysis^37^ and mesocosm experiments have shown that predation by flagellates and ciliates can structure the composition of bacterial communities^38^. Interestingly, a number of studies have reported that *Alphaproteobacteria* are resistant to predation (e.g., ref. 39), whereas others have indicated that members of the *Betaproteobacteria* are vulnerable to grazing (ref. 33 and references therein). We note that *Alphaproteobacteria* dominated *Sphagnum* interstitial water samples, while *Betaproteobacteria* dominated pool communities (Fig. 1).

Thirdly, we suggest that the distinction between *Sphagnum* interstitial water and pools communities may be linked to differences in connectivity within these two habitats. *Sphagnum* interstitial water communities are more likely to be interconnected and further linked to terrestrial communities through hydrological networks, favouring microbial dispersal. Dispersal can lead to increased local species richness because it allows new species to enter communities^18^, leading to higher gamma diversity and reduced community dissimilarity^17^, patterns we observed in *Sphagnum* interstitial water samples. Similar findings have been reported in previous studies for invertebrates^40^, plants^17^, and bacteria and viruses^41^, suggesting that this may be a universal phenomenon. In addition, *Sphagnum* interstitial water samples showed a greater proportion of *Acidobacteria* and *Gammaproteobacteria*, phyla commonly found in terrestrial communities^16^. In contrast, clear and vegetated pools can be seen as discrete, unconnected patches isolated from the surrounding soils, which allows for the development of more dissimilar communities, even if environmental conditions are similar, as those of vegetated pools (Fig. 5c). This concept may be supported by the increase in abundance and diversity of common freshwater phyla such as *Betaproteobacteria* and *Actinobacteria*^15^,^16^,^42^ in pool communities relative to *Sphagnum* interstitial water communities. Indirect mechanisms such as dispersal-mediated trophic interactions can also generate apparent patterns of dispersal limitation in aquatic metacommunities^43^.

In summary, this study showed that species sorting, as a result of both abiotic and biotic interactions, plays a pivotal role in explaining differences in microbial (general and putative nitrogen-fixing bacteria) community diversity and composition across peatland water habitats. In contrast, within habitat differences were better explained by the degree of microbial dispersal, which is higher in *Sphagnum* interstitial water communities. This has implications for conservation planning, as the high turnover in species composition in pool communities suggests that maximizing protected area is the key to maintaining diversity in the long term. It remains to be elucidated how these changes in microbial structure and composition will affect ecosystem function.

## Materials and Methods

### Study site, sampling and chemical analysis

Two peatlands from Tierra del Fuego Province (Argentina) separated by a distance of 50 km were sampled in February 2014: Andorra peat bog (AN), located in the Andorra Valley (54°45′ S; 68°20′ W) and Rancho Hambre peat bog (RH), in Tierra Mayor Valley (54°44′ S; 67°49′ W) (Supplementary Fig. 7). Both peatlands are raised, nutrient-poor ombrotrophic peat bogs^44^. Samples were collected at twelve sampling points along a transect crossing each peat bog dome (n = 24). The sampling points included three different habitat types (four replicates each): interstitial water from *Sphagnum magellanicum* matrix (SM), clear pools (CP) and vegetated pools (VP), with the latter two representing patches within the *Sphagnum* matrix. Vegetated pools showed prolific growth of *Sphagnum* cf. *fimbriatum* and in some instances *Drepanocladus uncinatus*, while clear pools showed a mud bottom mainly formed by decaying *S. magellanicum*. Pools were sampled at the margins, while interstitial water from the *Sphagnum* matrix was collected by aseptically squeezing the mosses *in situ*. Sampled water (250 ml) was filtered through 0.22-μm sterile nitrocellulose membranes (Nalgene, Rochester, NY, USA), and membranes placed in RNAlater (Sigma-Aldrich, St. Louis, Mo, USA) and stored at 4 °C until further processing.

pH and conductivity were measured *in situ* using a Hach Sension 156 multiparametric probe (Hach, Loveland, CO, USA). Nutrient (ammonium, phosphate, total N and total P) concentrations were measured using a Hach DR2800 spectrophotometer and their corresponding reagent kits, following standard methods described in the Hach DR2800 spectrophotometer procedures manual (www.hach.com). Ammonium and phosphate concentrations were determined according to the salicylate (No. 8155) and ascorbic acid (No. 8048) Hach methods, respectively. Total N and total P were determined by performing an acid digestion with potassium persulphate and boric acid^45^ followed by nitrate and phosphate determinations by the cadmium reduction (No. 8192) and ascorbic acid (No. 8048) Hach methods, respecrively. Dissolved organic carbon (DOC) was determined using the high temperature Pt catalyst oxidation method (Shimadzu analyser TOC-5000A, SM5310B technique) following the recommendations of Sharp *et al*.^46^. Chlorophyll *a* (Chl *a*) concentration corrected for phaeopigments was determined spectrophotometrically (for details see ref. 14). Metadata and water chemistry values are shown in Supplementary Table S2.

### DNA extraction and amplicon sequencing

DNA was extracted from half of each filter, cut into small pieces with a sterile blade, using a PowerSoil DNA isolation kit (MoBio Laboratories, Carlsbad, CA, USA). PCR was performed in a single-step PCR using HotStarTaq Plus Master Mix Kit (Qiagen, Valencia, CA) with primer pairs 515F (5′-GTGYCAGCMGCCGCGGRA-3′) and 909R (5′-CCCCGYCAATTCMTTTRAG-3′) for the 16S rRNA genes^47^, and nifH1F (5′-TGYGAYCCNAARGCNGA-3′) and nifH2R (5′-ADNGCCATCATYTCNCC-3′) for the nifH genes^48^. Triplicate PCR products were pooled after amplification, mixed in equal concentrations with samples targeting a specific gene and purified using Agencourt Ampure beads (Agencourt Bioscience Corporation, MA, USA). Sequencing was carried out on an Illumina MiSeq2000 using a paired-end approach^49^ at the Molecular Research LP next generation sequencing service (http://www.mrdnalab.com).

16S rRNA gene sequences were analysed in MOTHUR^50^, following a previously established pipeline^51^, using the Silva core set (http://www.arb-silva.de) for alignment. Reads were removed from further analysis if at least one of the following criteria was met: (i) reads shorter than 200 bp, (ii) number of ambiguous bases greater than 5, and (iii) presence of homopolymers with more than 8 bp. One mismatch to the sample-specific barcode and to the target-specific primer were allowed. After removal of chimeras and poor quality reads, a total of 3625057 sequences were obtained. Sequences were grouped into OTUs, defined using a 97% sequence similarity cut-off. Taxonomic affiliation was determined using the naive Bayesian rRNA classifier in MOTHUR; 80% confidence level. Singletons were removed and each sample was rarefied to 39908 sequences (the lowest number of sequences in any sample). A total of 21221 OTUs (957792 sequences) were retained for analysis.

Amplicons of the *nifH* genes were analysed using the FunGene pipeline (http://fungene.cme.msu.edu/FunGenePipeline/)^52^. Filtered reads were translated into amino acid sequences and clipped at 60 aa. Further analyses were carried out on amino acid sequences clustered at 95% similarity^53^. Representative sequences for each cluster were classified using the NCBI algorithm BLASTP. Singletons were removed and each sample was rarefied to 911 sequences. 327 OTUs (18220 sequences) from 20 samples, four samples did not give any amplification product, were kept for further analysis.

### Statistical analysis

OTU richness and diversity indices (Shannon, Inverse Simpson and Pielou’s evenness), together with rarefaction curves, Chao1 and Good’s coverage estimates were calculated using MOTHUR. We applied mixed model ANOVA to determine significant differences in diversity and water chemistry between habitat types using the phia package^54^. In these analyses, we specified peat bog ID as a random factor. Abiotic data were standardized and pair-wise distances computed based on Euclidean distances. The community data matrices was Hellinger-transformed and the Bray-Curtis distance measure was used to generate a dissimilarity matrix. Weighted UniFrac dissimilarities were also obtained^55^. The structure of the bacterial community and the environmental variables were visualized using non-metric multidimensional scaling. The effect of abiotic data in explaining variation in bacterial community structure was assessed by distance-based redundancy analysis after forward selection of the best set of parameters that could explain the variation in community composition. A permutational analysis of variance was used to test for differences in composition between habitats, ‘adonis’ function (strata = peat bog ID) in vegan for R; whereas permutation dispersion was used to test for differences in their within-habitat dissimilarity, ‘betadisper’ function.

We used LEfSe analysis^56^ to explore the presence of taxonomic groups that can serve as biomarkers for different classes (habitats). Statistically significant groups are reported with high LDA (Linear Discriminant Analysis) scores, which characterize the degree of consistency in relative abundance between features (genera) together with their effect relevance in each class. Correlations between the two biotic distance matrices were tested using the ‘mantel’ function in the ecodist package for R.

## Additional Information

**How to cite this article**: Oloo, F. *et al*. Habitat heterogeneity and connectivity shape microbial communities in South American petlands. *Sci. Rep*. **6**, 25712; doi: 10.1038/srep25712 (2016).

## Supplementary Material

Supplementary Information
