# Supplementary material for: Habitat heterogeneity and connectivity shape microbial communities in South American peatlands
Source: Sci Rep. 2016 May 10;6:25712. doi: 10.1038/srep25712 (PMC4861955; doi:10.1038/srep25712)

Habitat heterogeneity and connectivity shape aquatic microbial communities in South American peatlands.

Felix Oloo, Angel Valverde, María Victoria Quiroga, Surendra Vikram,
Don Cowan, and Gabriela Mataloni

Supplementary Table S1. Diversity measures and estimates.

Supplementary Table S2. Metadata and water chemistry of samples from pools and *S. magellanicum* interstitial waters.

Supplementary Figure S1. Rarefaction curves. (a) 16S rRNA genes (b) *nifH* genes.

Supplementary Figure S2. Diversity measures for the three habitats. The middle line in each box depicts the median of the respective data set. The box width represents 50% of the data, while both whiskers and outliers indicate the distribution of remaining data points, thus representing the overall variation. Different letters above each box denote a significant mean difference between respective habitats ($P < 0.05$). CP, clear pools; VP, vegetated pools; SM, *S. magellanicum* interstitial waters.

Supplementary Figure S3. Taxonomic affiliation of putative nitrogen-fixing bacteria obtained by BLAST analysis. CP, clear pools; VP, vegetated pools; SM, *S. magellanicum* interstitial waters.

Supplementary Figure S4. Multidimensional scaling diagrams showing the degree of similarity (weighted UniFrac) between (a) total bacterial communities, (b) putative nitrogen-fixing communities.

Supplementary Figure S5. Differences in within-habitat dissimilarity (Bray-Curtis index) measured by permutation dispersion. Different letters above each box indicate statistically significant differences in means between (a) bacterial communities and (b) environmental data ($P < 0.05$). CP, clear pools; VP, vegetated pools; SM, *S. magellanicum* interstitial waters.

Supplementary Figure S6. Differences in environmental conditions. Different letters above each box denote a significant mean difference between respective habitats ($P < 0.05$). CP, clear pools; VP, vegetated pools; SM, *Sphagnum magellanicum* interstitial waters.

Supplementary Figure S7. Maps of the sampling area. AN, Andorra peat bog; RH, Rancho Hambre peat bog. CP, clear pools; VP, vegetated pools; SM, *S. magellanicum* interstitial waters. The maps were generated using Google Earth Pro v7.0 (https://www.google.com) and CorelDRAW vX8 (http://www.coreldraw.com). Map data: Google, DigitalGlobe.

Supplementary Table S1.

Total bacteria

Sample	Location	Habitat	Richness	Shannon	Inverse Simpson	Pielou's	Chao1	Good's coverage
ANCP1	AN	CP	2210	5.6	62.8	1277.5	4570.3	98%
ANCP2	AN	CP	1962	4.4	17.4	1321.4	7738.4	97%
ANCP3	AN	CP	1327	4.2	21.9	930.5	4530.6	98%
ANCP4	AN	CP	2432	4.1	10.0	1717.4	4642.2	97%
ANSM1	AN	SM	3423	5.8	63.0	1953.3	6256.6	96%
ANSM2	AN	SM	3370	5.7	69.7	1941.5	6550.6	95%
ANSM3	AN	SM	3459	5.7	64.8	1987.7	6717.5	95%
ANSM4	AN	SM	3182	5.5	59.3	1857.8	6534.6	96%
ANVP1	AN	VP	2837	5.4	36.2	1686.8	5149.2	96%
ANVP2	AN	VP	2000	3.5	8.2	1593.9	4097.5	97%
ANVP3	AN	VP	2762	4.5	12.6	1824.8	5061.4	97%
ANVP4	AN	VP	2980	5.3	28.9	1793.5	5494.0	96%
RHCP1	RH	CP	1668	4.0	19.3	1200.6	3796.8	98%
RHCP2	RH	CP	1974	3.9	19.9	1442.0	4220.4	97%
RHCP3	RH	CP	1412	4.0	16.1	1019.5	3110.8	98%
RHCP4	RH	CP	1179	4.6	18.9	777.5	2648.7	98%
RHSM1	RH	SM	4010	5.8	44.2	2275.7	7242.0	95%
RHSM2	RH	SM	1761	5.7	79.0	1007.8	3712.2	98%
RHSM3	RH	SM	3123	6.1	82.1	1733.1	5140.5	97%
RHSM4	RH	SM	1753	5.9	121.8	985.4	3795.3	98%
RHVP1	RH	VP	868	3.1	13.5	760.1	4129.1	98%
RHVP2	RH	VP	2481	3.6	7.7	1951.8	5220.6	96%
RHVP3	RH	VP	1019	5.0	79.5	629.6	3217.8	98%
RHVP4	RH	VP	2560	4.1	13.6	1814.6	5215.3	96%

Nitrogen-fixing bacteria

Sample	Location	Habitat	Richness	Shannon	Inverse Simpson	Pielou's	Chao1	Good's coverage
ANCP1	AN	CP	17	2.0	4.7	25.1	17.0	100%
ANCP2	AN	CP	14	1.2	2.2	95.5	14.0	100%
ANCP3	AN	CP	13	1.6	3.7	26.5	13.0	100%
ANCP4	AN	CP	64	3.1	12.7	56.1	64.0	100%
ANSM1	AN	SM	89	3.6	19.8	69.1	89.0	100%
ANSM2	AN	SM	74	3.1	8.7	65.1	74.0	100%
ANSM3	AN	SM	48	3.0	10.5	44.1	48.0	100%
ANSM4	AN	SM	49	2.9	10.1	45.6	49.0	100%
ANVP1	AN	VP	81	3.4	12.6	66.9	81.0	100%
ANVP2	AN	VP	4	0.9	2.1	61.3	4.0	100%
ANVP3	AN	VP	13	1.9	5.6	19.5	13.0	100%
ANVP4	AN	VP	69	2.6	6.0	72.0	112.5	97%
RHCP1	RH	CP	23	1.7	3.6	42.1	23.0	100%
RHCP2	RH	CP	12	2.3	7.7	14.6	12.0	100%
RHCP3	RH	CP	NA	NA	NA	NA	NA	NA
RHCP4	RH	CP	NA	NA	NA	NA	NA	NA
RHSM1	RH	SM	89	3.7	27.2	67.4	320.0	98%
RHSM2	RH	SM	44	2.6	6.6	46.7	48.2	99%
RHSM3	RH	SM	27	1.6	3.0	58.3	27.0	100%
RHSM4	RH	SM	8	1.1	2.0	80.4	8.0	100%
RHVP1	RH	VP	NA	NA	NA	NA	NA	NA
RHVP2	RH	VP	11	1.9	4.8	16.5	11.0	100%
RHVP3	RH	VP	NA	NA	NA	NA	NA	NA
RHVP4	RH	VP	46	2.4	5.3	52.8	166.0	98%

NA: no amplification

Supplementary Table S2.

Barcode		Sample	Location	Habitat	Latitude	Longitude	pH	Conductivity	NH_4	Total N	Total P	Chl a	DOC
16s rRNA	nifH							µS/cm	µg/L	µg/L	µg/L	µg/L	mg/ml
TCCTGTAC	AGAGCTATT	ANCP1	AN	CP	54.75611	68.33778	4.62	34	50	7900	220	4.0	15.12
TCCTTCCA	AGAGTACGT	ANCP2	AN	CP	54.75543	68.33779	4.4	39.7	30	10800	110	1.4	11.91
TCCTTCTT	AGAGTGCCT	ANCP3	AN	CP	54.75516	68.33839	3.6	38.9	10	4100	520	6.1	14.49
TCGAAAGG	AGATATACT	ANCP4	AN	CP	54.7533	68.33976	4	9.7	40	8500	200	5.9	12.23
TCGACCGG	AGCATTACT	ANSM1	AN	SM	54.75586	68.33755	3.95	122.1	150	100	1070	10.1	23.04
TCGACCTA	AGCCACGCT	ANSM2	AN	SM	54.7546	68.33848	3.86	144.4	70	6400	620	29.5	22.83
TCGACGAA	AGCCACGGT	ANSM3	AN	SM	54.75367	68.33915	3.8	67.6	10	2300	520	28.9	16.12
TCGACGAC	AGCCATCGT	ANSM4	AN	SM	54.7531	68.3396	3.3	140.1	30	900	1150	25.3	20.35
TCGAACCT	AGATGATGT	ANVP1	AN	VP	54.75539	68.33811	4.28	46.8	20	2700	550	4.4	11.61
TCGAAGAG	AGCACGACT	ANVP2	AN	VP	54.75511	68.33801	4.3	31.1	10	2900	410	3.5	8.421
TCGAATCC	AGCAGAACT	ANVP3	AN	VP	54.75406	68.33896	4.6	23.2	20	2900	420	1.3	6.285
TCGACAAC	AGCATGCCT	ANVP4	AN	VP	54.7533	68.33976	4.1	33.2	30	4900	260	7.1	9.363
TCCGGCCG	ACTTCACTT	RHCP1	RH	CP	54.74796	67.82478	4.26	21.7	40	3800	910	2.5	7.993
TCCGGTAA	ACTTCTATT	RHCP2	RH	CP	54.74689	67.82554	4.67	25.8	10	1900	160	0.3	6.783
TCCGTCTT	ACTTGCAGT	RHCP3	RH	CP	54.74512	67.82451	6.19	22	0	2500	210	8.2	5.077
TCCGTGCG	ACTTGGTGT	RHCP4	RH	CP	54.74432	67.8242	4.35	30.2	20	1900	270	10.1	12.85
TCCTCCCT	AGACTGCTT	RHSM1	RH	SM	54.74387	67.82494	3.57	74.7	150	1800	750	15.2	24.1
TCCTCGTA	AGACTTACT	RHSM2	RH	SM	54.74778	67.8249	4.37	61.6	80	6400	1920	15.5	19.37
TCCTCGTG	AGAGCAAGT	RHSM3	RH	SM	54.74627	67.82583	3.93	85.4	120	3400	850	11.9	28.78
TCCTGGCA	AGAGCGCGT	RHSM4	RH	SM	54.74487	67.82455	3.94	69.4	20	3300	510	2.6	24.59
TCCGTGCT	AGAACGCTT	RHVP1	RH	VP	54.74914	67.82542	4.36	41.4	0	2000	220	1.8	16.89
TCCGTTCC	AGAACTATT	RHVP2	RH	VP	54.74809	67.82444	4.36	37.1	20	1800	300	1.6	15.87
TCCGTTTT	AGAATGTGT	RHVP3	RH	VP	54.74465	67.82464	3.93	47.9	10	1400	470	1.6	21.23
TCCTAATA	AGACAACTT	RHVP4	RH	VP	54.74394	67.82448	4.44	48.6	0	1500	320	3.8	18.12

AN, Andorra peat bog; RH, Rancho hambre peat bog, CP, clear pools; VP, vegetated pools; SM, *Sphagnum magellanicum* interstitial waters

Supplementary Figure S1.

a

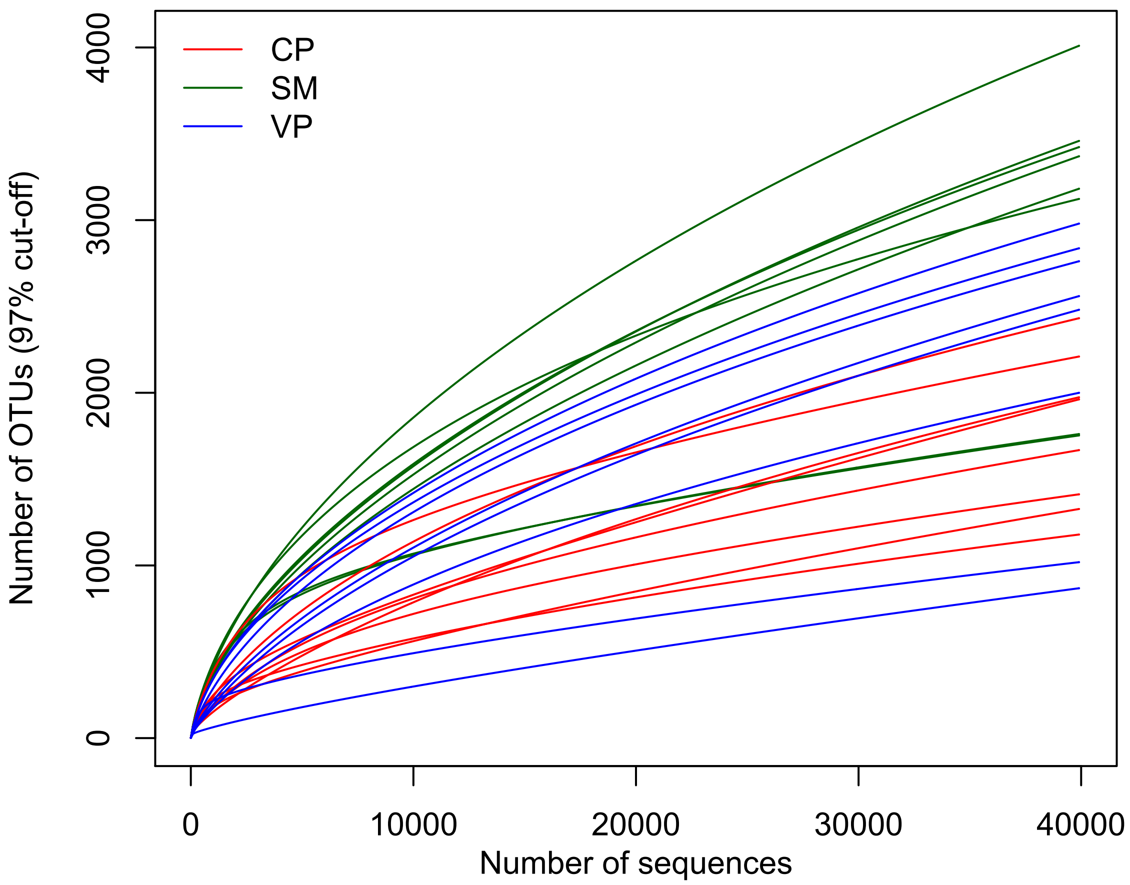

b

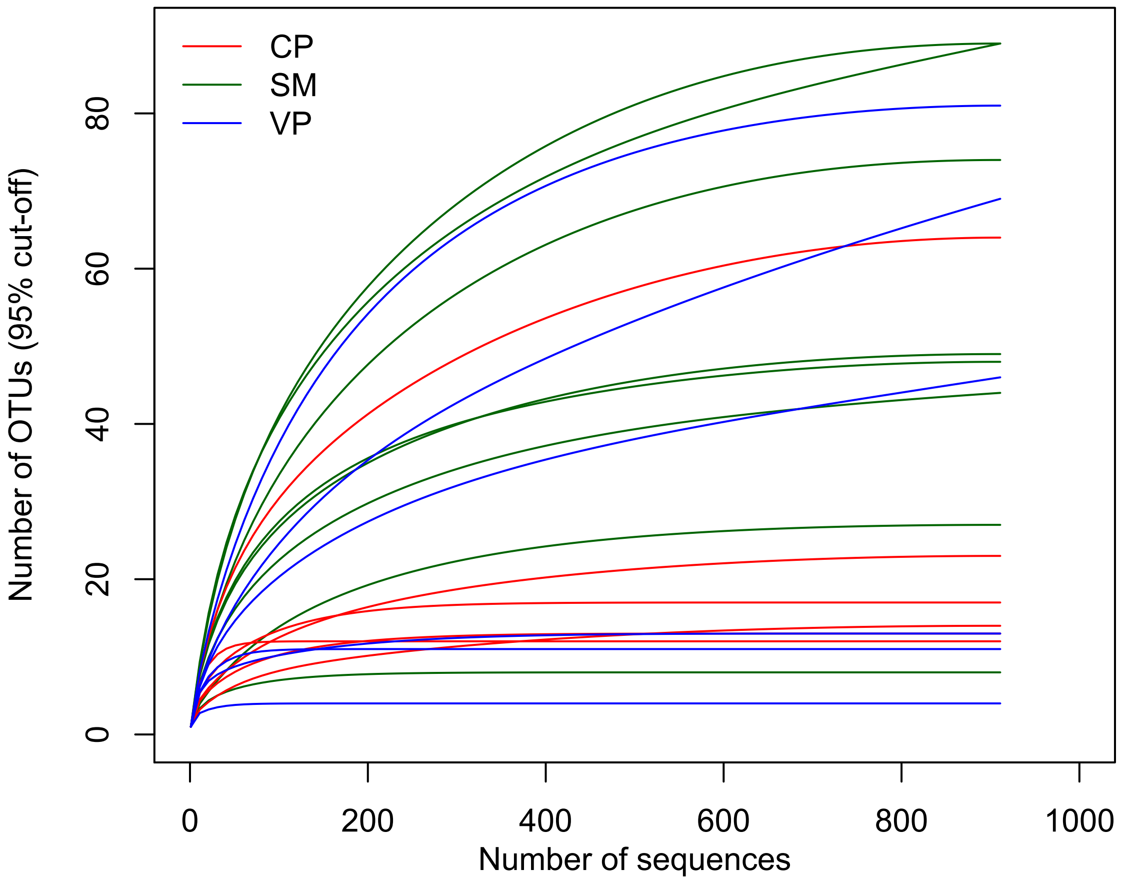

Supplementary Figure S2.

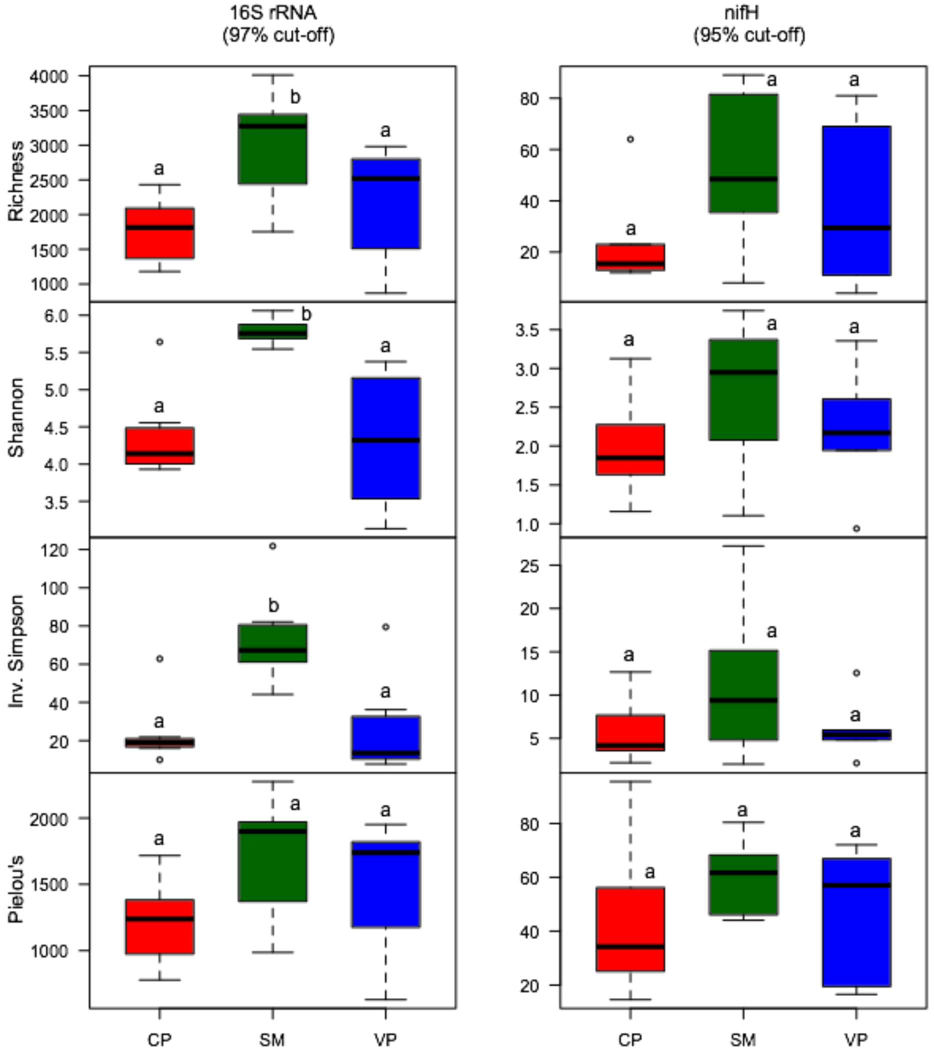

Supplementary Figure S3.

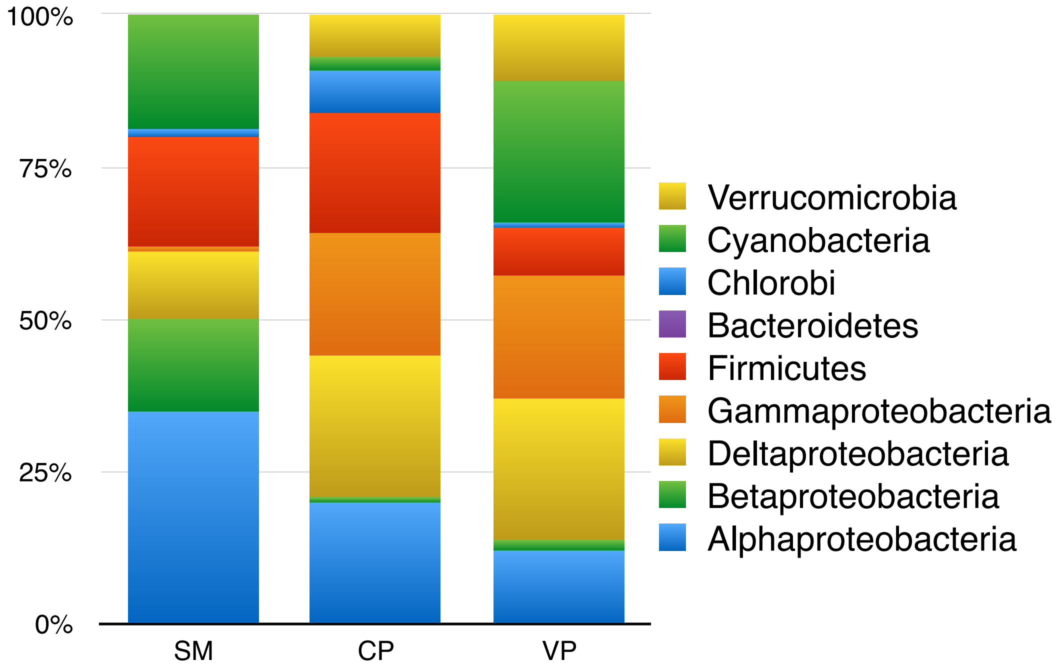

Supplementary Figure S4.

a

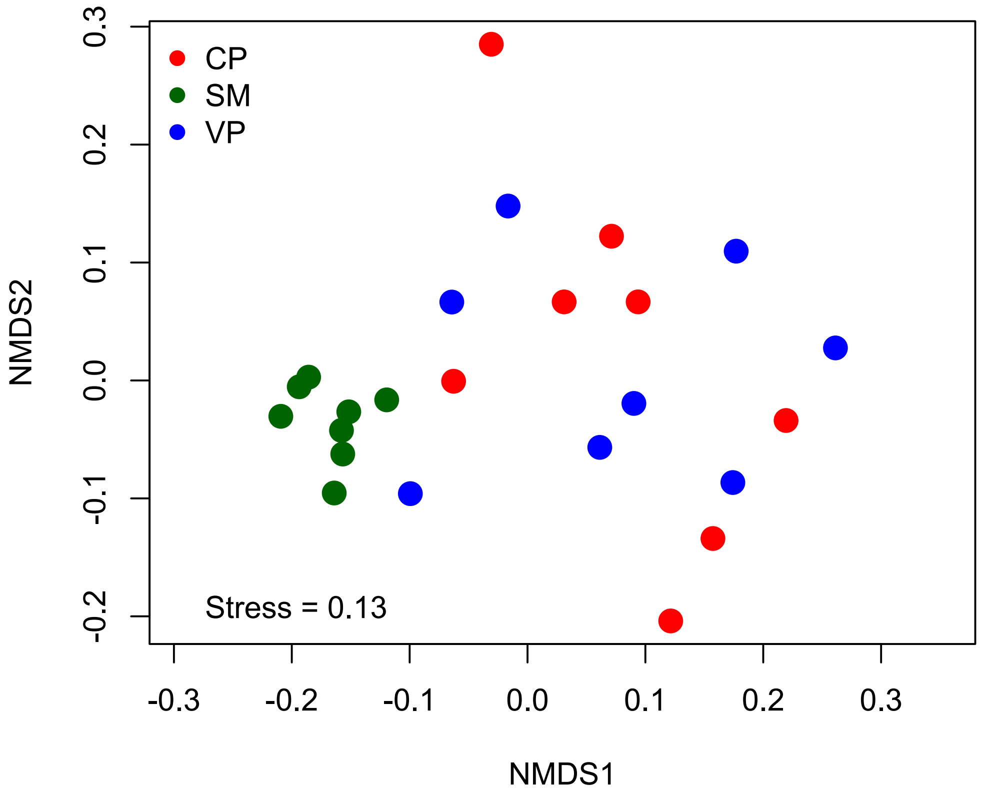

b

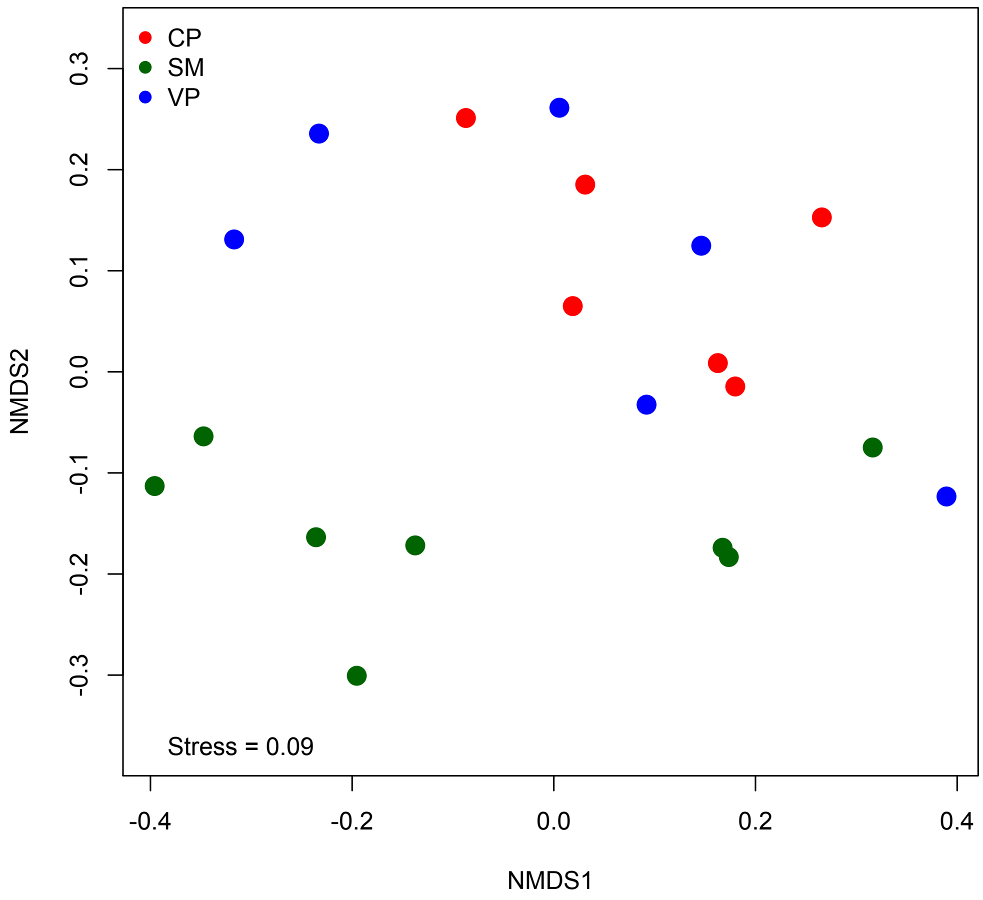

Supplementary Figure S5.

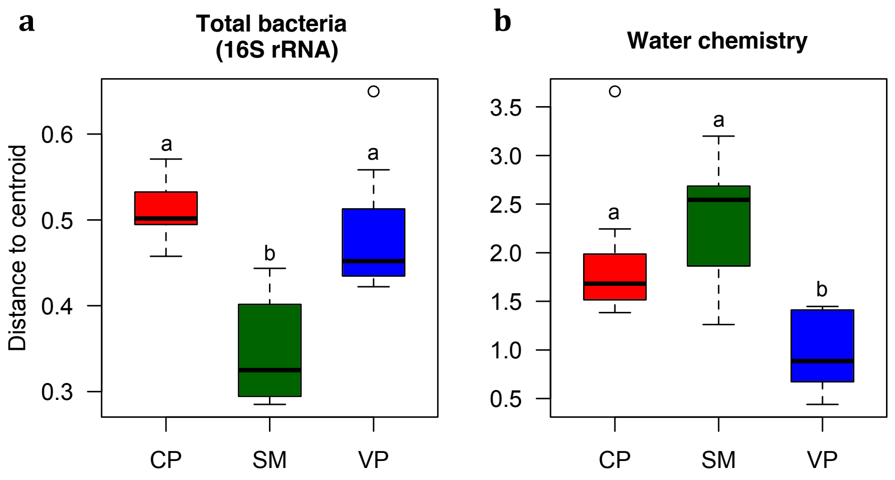

Supplementary Figure S6.

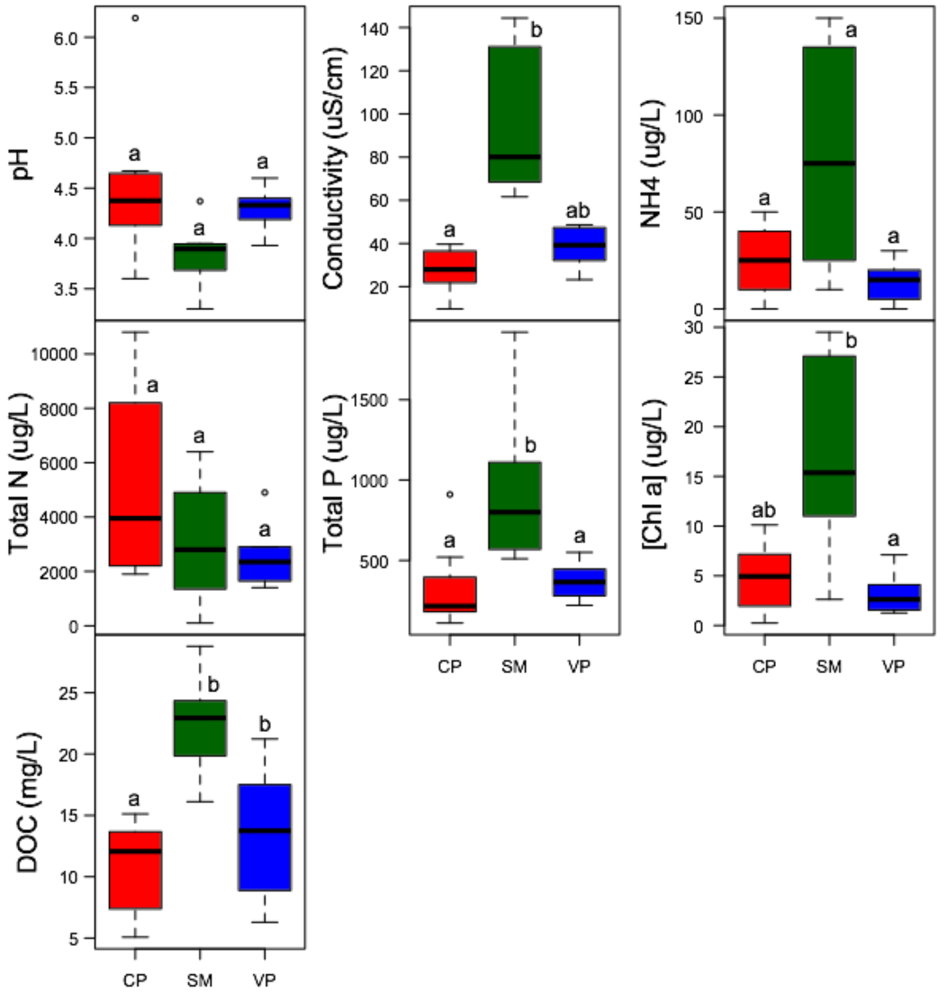

Supplementary Figure S7.

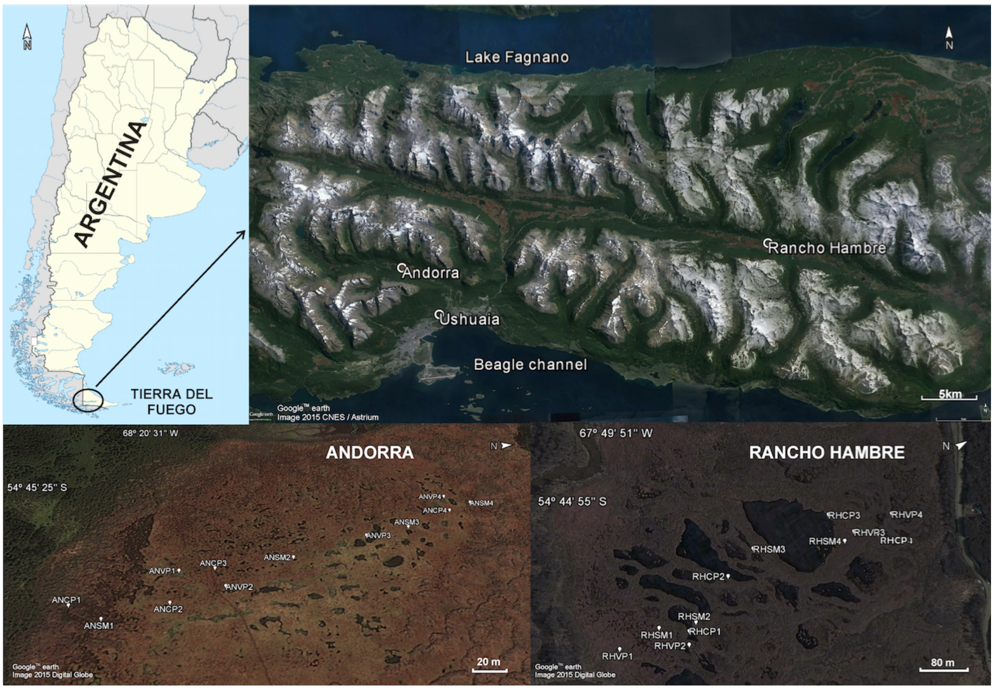

Supplement: Supplementary Information [file srep25712-s1.pdf]
